# The morbidity and the spectrum of applied drugs in shelter cats in the Czech Republic

**DOI:** 10.3389/fvets.2022.1025197

**Published:** 2022-12-05

**Authors:** Veronika Vojtkovská, Michal Kaluža, Eva Voslářová, Vladimír Večerek, Lenka Tomečková

**Affiliations:** ^1^Department of Animal Protection and Welfare and Veterinary Public Health, University of Veterinary Sciences Brno, Brno, Czechia; ^2^Department of Public Veterinary Medicine and Animal Welfare, The University of Veterinary Medicine and Pharmacy in Košice, Košice, Slovakia

**Keywords:** shelter cat, morbidity, treatment, drugs, length of stay

## Abstract

Maintaining appropriate living conditions and good health of the animals should be one of the main priorities of cat shelters. The aim of this study was to assess the morbidity of shelter cats on the basis of analysis of the shelter health records in terms of the nature and quantity of medicaments and other supportive products administered in two no-kill shelters in the Czech Republic. The subject of the study were the health records of 1,884 cats, which were admitted to the selected shelters from 1.1.2013 to 31.12.2021 and their stay in the shelter was terminated in the monitored period. More than half of all cats whose records were analyzed had at least one health record made during their stay in the shelter. The overall length of stay of cats in the shelter (regardless of the outcome) in which one or more health records were found was significantly longer compared to the length of stay of cats without any health records. The highest number of health records was found in kittens <6 months old. Antibiotics, antiparasitics, and complementary/protective substances were the most used among all administered drugs. Broad-spectrum antibiotics have been administered to cats most often. In terms of classification of antibiotics according to the active substance, the most used antibiotics were penicillins, tetracyclines, and fluoroquinolones. Antibiotics were mostly used to treat diseases related to the upper and lower respiratory tract and their symptoms and gastrointestinal problems. The findings of the study contribute to understanding health problems and approaches to treating the shelter cats.

## Introduction

Shelters represent facilities with a high concentration and dynamics of animals, which, from an epidemiological point of view, is a predisposing factor to the development of health disorders (1). Similarly, from an ethological point of view, the increased concentration of animals is a detriment to the development of good living conditions in species normally living primarily solitary lives. For many cats, the shelter environment is very stressful (2) due to the exposure to the new stimuli and the overall lack of control over the environment (3). Stress significantly increases animal morbidity, weakens the immune system (4) and is associated with the development of gastrointestinal diseases (5). Stress also affects the development of idiopathic cystitis (6) and dermatological problems (7). A link was also found between inactivity and the development of diabetes and obesity (8, 9); stress in cats often manifests in the form of inactivity (7). A deterioration in at least one of the monitored health indicators over the stay at a shelter was found in as many as 41.6% of cats (10).

The level of morbidity in a shelter is related to two groups of factors. Firstly, aspects that can be regulated to a certain extent as they are related to the environment of the shelter (type of housing for cats, its division and equipment) and the application of disease control measures (preventive sanitary strategies leading to the effective removal of pathogens from the environment, placing admitted individuals in quarantine, isolation of individuals with symptoms of the disease, level of veterinary care provided). Maintaining appropriate living conditions of the population should be one of the main priorities of shelters, as good animal health is a factor that increases adoption potential (11–13) and shorten length of stay in the shelter. The quality of the veterinary care provided in the shelter is related to the capacity of the shelter, the type of shelter, its financial capabilities, the presence of qualified staff, equipment and other factors; its level may therefore vary significantly among facilities (14). A need to decide whether and under what circumstances to provide an animal with costly care or whether euthanasia is more viable, especially considering other animals in need, is in practice a common issue.

The second group of factors related to morbidity includes factors that can be influenced by the facility only to a small extent because these are related to the history and condition of the individuals admitted. As we reported in our recent study (10), a deterioration in health concerned as many as 54.5% of cats admitted to the shelter. Marston and Bennett (15) reported half of all animals admitted to the shelter in Australia showed symptoms of upper respiratory tract infection, 30% of cats admitted were not in optimal body condition and 10.9% of cats had older or newer injuries present.

The task of the shelter veterinarian is to choose the most effective treatment for the animals, taking into account the risk of endangering the entire population of the shelter animals in the event of the introduction of the disease. The choice of a particular medicinal product should also be preceded by consideration of possible side effects, costs (16) and the least stressful route of administration. If antibiotics are used, the possibility of the development of antibiotic resistance should also be taken into account (17).

The aim of this study was to assess the morbidity of shelter cats on the basis of analysis of the shelter health records in terms of the nature and quantity of medicaments and other supportive products administered in no-kill shelters in the Czech Republic.

## Materials and methods

### Study site

For the purposes of the study, cooperation was established with two private no-kill shelters with group housing of cats in the Czech Republic. Facilities were selected on the basis of the method of keeping records – in the Czech Republic, facilities providing care to stray and abandoned animals do not incur a legal obligation to keep records regarding the health condition of animals and administered medicinal products, therefore, a considerable number of establishments do not keep such records. The facilities were also selected taking into account the similarities in management and the way the animals are housed. The capacity of shelters was 100 (Shelter A) and 25 animals (Shelter B) in the monitored period. In both shelters, the animals were housed in a group (in Shelter B, cats were housed in a single group of animals, in Shelter A, there were several groups of cats). Both shelters are set up in a residential area, Shelter A is operated in a private house (206 m^2^), Shelter B in a private apartment (38 m^2^). In both facilities, in addition to the interior, the animals also have access to a roofed outdoor enclosure with dimensions of 24 m^2^ (Shelter A) and 15 m^2^ (Shelter B). The total floor area per animal is 2.3 m^2^ in Shelter A and 2.1 m^2^ in Shelter B. Upon admission to the shelters, the cats are placed separately (possibly also in a pair or group if they come from the same environment) in quarantine boxes. The duration of quarantine depends on the health status and history of each cat, however, it does not last <5 days in any individual. While in quarantine, the animals are inspected by a veterinarian and the basic veterinary procedures are performed (vaccination, internal and external parasite treatment if necessary). Admitted animals are also microchipped and, when their health and age permit, neutered. Neither shelter has its own veterinary facility, therefore, the animals are transported to a contracted veterinary clinic for veterinary treatment. In both shelters, animals are tested upon admission for the presence of antibodies against feline immunodeficiency virus (FIV) and feline leukemia virus (FeLV). In Shelter A, the animal care is provided by one caregiver and three volunteers, and in Shelter B by one permanent caregiver and one additional volunteer. In both shelters, the animals are fed *ad libitum* with dry, granulated food of super-premium quality supplemented with wet, canned food of various brands. In both shelters, the animals share bowls for food and water; they are placed evenly throughout the individual rooms of the shelters so that each animal can access them. The rooms used for housing cats are equipped with a number of enrichment elements in the form of toys, cat trees and elevated places with spots for hiding. In Shelter A, there are 32 cat toilets of open and closed type, Shelter B has eight cat toilets of closed type at its disposal (in both shelters there is one toilet per ~three cats). Toilets are cleaned daily or as needed. The flooring of the shelters is cleaned on a daily basis using common disinfectants.

### Health records

The subject of the study were the health records of 1,884 cats, which were admitted to the selected shelters from 1.1.2013 to 31.12.2021 and their stay in the shelter was terminated in the monitored period. The records provided by the shelters included basic information on the sex and age of the animals (if the age of an animal was unknown, the approximate age was determined by the shelter caregiver on admission), the date of admission to the shelter, the date of end of the stay at the shelter, the outcome (adoption, unassisted death, euthanasia, return to the owner, return to the capture site, transfer to another facility, displacement of the animal), records related to the treatment (name of the medicinal product, administration count, reason for administration) and the observed symptoms of the deteriorated health of the cats. The records did not include information on surgical procedures and treatment given to cats at veterinary clinics in case of their hospitalization. In order to assess the impact of the length of stay (LOS) of cats in the shelter on the number of medical records, the LOS of each cat was calculated in days (LOS represented the difference between the cat's intake date and the date when its stay in the shelter was terminated). With regard to the analysis of age as a possible factor affecting the morbidity of cats, animals were classified into 4 categories by age (kittens: ≤6 months, young cats: 6 < x ≤ 12 months, adult cats: 1 < x ≤ 8 years, old cats: >8 years).

For the purpose of the analysis of the type of antibiotics administered, they were categorized according to their effect into broad-spectrum, medium to broad-spectrum and narrow-spectrum. The categorization of antibiotics within this paper is based on their classification according to the extent of effect on bacterial flora specified in the Summary of Product Characteristics (SPC), which is available for each authorized veterinary or human medicinal product (18). In addition, antibiotics were also categorized according to their chemical structure into amphenicols, cephalosporins, fluoroquinolones, lincosamides, macrolides, nitroimidazoles, penicillins, aminoglycosides, potentiated sulfonamides and tetracyclines (19).

Analyses of the number of administrations of antiparasitic products included only administrations beyond routine deworming after admission to the shelter (this administration was made for the purpose of eliminating internal parasites). The shelters did not perform a coprological examination of feces upon admission of each animal, a medicament against endoparasites was preventively administered to all admitted animals. Similar to the administration of products against endoparasites, routine vaccinations and administrations of drugs to perform euthanasia were not included in the analysis of the nature and quantity of medicaments and other supportive products. Cats that underwent only vaccination, deworming and neutering, and for which no other health record was found were considered healthy and in the reporting of the results were designated as animals with no health records.

### Statistical analysis

The data were analyzed using the statistical software Unistat 6.5 for Excel (Unistat Ltd., UK). The normality of the data was verified by the Shapiro-Wilk test (irregular distribution was found). The Chi-square test was used to analyze differences in the number of animals in categories of basic variables (sex, age, method of termination of stay in the shelter). This test was also used for other analyses, which involved comparing the number of animals in the created categories, or the number of health records and medications. The Mann-Whitney U test was used to verify the impact of sex on the LOS of cats in the shelter and also to monitor differences in LOS in the shelter until adoption in cats without health records and with one or more health records within individual age categories. In the case of LOS analyses for variables with multiple categories, the Kruskal-Wallis ANOVA was used. Subsequently, when the effect of the variable was significant, a non-parametric Tukey-type test was used as a *post-hoc* test for pairwise comparisons. The correlation between the overall LOS or LOS in the shelter until adoption and the number of health records was verified by Spearman's correlation coefficient. The value of *p* ≤ 0.05 was considered statistically significant.

## Results

### Morbidity of the monitored shelter cat population

A total of 1,884 cats were admitted to the selected shelters and also ended their stay during the monitored period. The mean LOS of cats in the shelter was 69.4 days, the median 39 days, the minimum 0 days, and the maximum 1,348 days. Table 1 summarizes the statistical differences in LOS and the number of cats admitted to the shelter according to their sex, age and outcome.

**Table 1:** Number and LOS of cats admitted to the shelter according to their sex, age and outcome.

	**Number of cats (%)**	**LOS in days (median)**
Sex	*p* = 0.4339^#^	*p* = 0.0504^&^
Females	955 (50.7)^a^	39.5^a^
Males	929 (49.3)^a^	37.0^a^
Age	*p* = 0.0000^#^	*p* = 0.7113^&^
≤6 months	1,120 (59.5)^a^	39^a^
6 < x ≤ 12 months	236 (12.5)^c^	37^a^
1 < x ≤ 8 years	481 (25.5)^b^	38^a^
>8 years	47 (2.5)^d^	34^a^
Outcome	*p* = 0.0000^#^	*p* = 0.0000^&^
Adopted	1,540 (81.7)^a^	42^ac^
Death (unassisted or euthanasia)	266 (14.1)^b^	23.5^b^
Returned to owner	42 (2.2)^c^	7^e^
Returned to capture site	31 (1.6)^c^	16^bd^
Relocated	2 (0.1)^d^	20.5^bcde^
Lost	3 (0.2)^d^	63^abcd^

a,b,c,d,e Percentages and medians within a column and the same variable with different superscript letters differ significantly (*p* < 0.05).

# Chi-square test.

& Kruskal-Wallis ANOVA (Mann-Whitney U test for sex).

Out of 1,884 cats, no health record was found in 727 cats (38.6%). In a significantly (*p* < 0.01) higher number of cats (1,157, 61.4%), one or more health records were found. The overall LOS of cats in the shelter (regardless of their outcome) in which one or more health records were found was significantly (*p* < 0.01) longer (median 51 days) compared to the LOS of cats without any health records (median 26 days). The LOS of adopted cats in which no health record was found was significantly (*p* < 0.01) shorter (median 28 days) compared to the LOS of adopted cats with one or more health records (median 54 days). The overall LOS of cats in the shelter significantly correlated (r_s_ = 0.3894, *p* < 0.01) with the number of health records, the significant correlation (r_s_ = 0.4092, *p* < 0.01) was found also between the number of health records and the LOS of adopted cats.

A total of 4,389 health records were made in the monitored cat population. Among these, 3,722 records (84.8%) concerned the administration of a medicaments and other supportive products, 663 records (15.2%) described a specific condition or indicator of a deteriorated health (e.g., the presence of diarrhea). The difference in number between cats that were given a medicaments or other supportive products at least once (1075) and the number of cats not given any such product (82) was significant (*p* < 0.01). Table 2 summarizes the numbers of cats that received a medicaments or other supportive products one or more times. The maximum number of administrations recorded per animal was 23, the mean number of administrations per cat was 3.2.

**Table 2:** Number of cats given a medicaments and other supportive products.

**Number of administrations of medicaments and other supportive products**	**Number of cats**	
	***n***	**%**
1	314	29.2^a^
2	256	23.8^b^
3	135	12.6^c^
4	83	7.7^d^
5	69	6.4^d^
6	74	6.9^d^
7	44	4.1^e^
8	30	2.8^e^
9	15	1.4^f^
10	11	1.0^f^
>10	44	4.1^e^
Total	1,075	100.0

a,b,c,d,e,f Chi-square test - percentages within a column with different superscript letters differ significantly (*p* < 0.05).

The results of the comparison of the number of cats without records and cats with health records across four age categories are shown in Table 3. A significant difference in the number of cats without health records and those with health records was found only in the youngest and oldest age category.

**Table 3:** Comparison of the number of cats without records and with health records across four age categories.

**Age category**	**Number of cats without health records**		**Number of cats with one or more health records**	
	***n***	**%**	***n***	**%**
≤6 months	343	47.18^a^^,^^y^	777	67.16^a^^,^^x^
6 < x ≤ 12 months	124	17.06^c^^,^^x^	112	9.68^c^^,^^x^
1 < x ≤ 8 years	246	33.84^b^^,^^x^	235	20.31^b^^,^^x^
>8 years	14	1.93^d^^,^^y^	33	2.85^d^^,^^x^
Total	727	100.00	1,157	100.00

a,b,c,d Chi-square test - percentages in the same column with different superscript letters differ (*p* < 0.05).

x,y Chi-square test - percentages in the same row with different superscript letters differ (*p* < 0.05).

Table 4 shows the results of the comparison of the LOS of different age categories of cats with regard to the number of health records found. In cats over 8 years of age, the LOS until adoption did not differ between animals for which no health record was found and animals with one or more health records. In all other age categories, a significant difference in LOS until adoption was found (*p* < 0.05).

**Table 4:** Comparison of the LOS of different age categories of cats for which no health records were made and cats for which one or more health records were made.

**Age category**	**Number of cats without health records**	**Number of cats with one or more health records**
	**LOS until adoption (median)**	**LOS until adoption (median)**
≤6 months	26^a^^,^^w^	53^b^^,^^w^
6 < x ≤ 12 months	28^a^^,^^wx^	54^b^^,^^wx^
1 < x ≤ 8 years	30^a^^,^^x^	67^b^^,^^x^
>8 years	37.5^a^^,^^wx^	67^a^^,^^wx^

a,b Mann- Whitney U test - percentages in the same row with different superscript letters differ (*p* < 0.05).

w,x Kruskal- Wallis ANOVA – LOS until adoption in the same column with different superscript letters differ (*p* < 0.05).

When comparing the LOS until adoption in animals of different ages without health records, a significant difference was found only between the category of the youngest cats (≤6 months) and adult cats (1 < x ≤ 8 years). In animals with one or more records, the result was similar (the youngest cats remained in the shelter significantly shorter than adult animals).

Among the monitored age categories of cats, the highest number of health records was found in cats younger than 6 months, similarly, the number of administrations of the medicaments and other supportive products was the highest in this category of cats (Table 5).

**Table 5:** Impact of age on the number of all health records found and on the number of administrations of medicaments and other supportive products.

**Age category**	**Number of all health records**		**Number of health records related to the administration of medicaments and other supportive products**	
	***n***	**%**	***n***	**%**
≤6 months	3,163	72.07^a^	2,705	72.68^a^
6 < x ≤ 12 months	337	7.68^c^	283	7.60^c^
1 < x ≤ 8 years	806	18.36^b^	674	18.11^b^
>8 years	83	1.89^d^	60	1.61^d^
Total	4,389	100.00	3,722	100.00

a,b,c,d Chi-square test - percentages in the same column with different superscripts differ (*p* < 0.05).

### Spectrum of medicaments and other supportive products administered to the monitored cat population in the shelter

During the monitored period, a total of 112 different products with a therapeutic or supportive effect containing a total of 127 active or supportive substances were administered to the monitored population of cats in the shelter. Based on the mechanism of action and the active substance, the products were categorized into 22 groups (Table 6).

**Table 6:** Number of administrations of medicaments and other supportive products used to treat the monitored cat population during the stay in the shelter.

**Category of products (number of products included)**	**Number of administrations of the product**	
	***n***	**%**
Antibiotics (42)	2,552	68.57^a^
Antibiotics in combination with corticosteroids (6)	67	1.80^f^
Antibiotics in combination with corticosteroids and other active substances (3)	70	1.88^f^
Corticosteroids (6)	31	0.83^g^
Antiparasitics (9)	284	7.63^b^
Antifungals (4)	53	1.42^f^
Antivirals (1)	5	0.13^hi^
Non-steroidal anti-inflammatory drugs (NSAIDs) (7)	101	2.71^e^
NSAID in combination with other active substances (1)	1	0.03^i^
Analgesics and spasmolytics (2)	8	0.21^h^
Complementary and protective substances (8)	194	5.21^cd^
Choleretics (1)	169	4.54^d^
Antiemetics (2)	102	2.74^e^
Antidiarrheals (2)	7	0.19^hi^
Laxatives (1)	1	0.03^i^
Diuretics (1)	5	0.13^hi^
Vasodilators (2)	7	0.19^hi^
Anticoagulants (1)	1	0.03^i^
Antihypertensives^1^ (2)	2	0.05^hi^
Anticonvulsants and anxiolytics (2)	2	0.05^hi^
Selected products for immunomodulation^2^ (6)	55	1.48^f^
Special treatment^3^ (3)	5	0.13^hi^
Total (112)	3,722	100.00

a−i Chi-square test - percentages in the same column with different superscript letters differ (*p* < 0.05).

1 Eye beta-blockers (1; 0.03%); angiotensin-converting enzyme inhibitors (1; 0.03%).

2 Immunoglobulins (15; 0.40%); immunostimulants (19; 0.51%); interferons (18; 0.48%); antihistamines (3; 0.08%).

3 Treatment of incontinence (1; 0.03%); treatment of hyperthyroidism (1; 0.03%); treatment of neurological problems (3; 0.08%).

Antibiotics were the most commonly used among all administered medicaments. Of the 1,157 cats in which at least one health record was found, an antibiotic, an antibiotic in combination with corticosteroids or an antibiotic with corticosteroids and other active substances were administered at least once to 1,023 animals (96.9%) (Table 7). The highest number of antibiotic administrations in one animal was 17, the mean number of administrations per cat was 2.6.

**Table 7:** Number of cats given an antibiotic, an antibiotic in combination with corticosteroids or an antibiotic with corticosteroids and other active substances.

**Number of antibiotic administrations**	**Number of cats**	
	***n***	**%**
1	388	37.93^a^
2	274	26.78^b^
3	133	13.00^c^
4	74	7.23^de^
5	56	5.47^e^
>5	98	9.58^d^
Total	1,023	100.00

a−e Chi-square test - percentages in the same column with different superscript letters differ (*p* < 0.05).

As opposed to other purposes, antibiotics were used to a statistically greater extent (*p* < 0.01, 52.2%) to treat diseases of the upper and lower respiratory tract and their symptoms (eye discharge, conjunctivitis, nasal discharge, breathing difficulties, visibility of the third eyelid, presence of pathological sounds related to breathing – cough, sneezing). 28.4% of antibiotics were administered for the treatment of gastrointestinal problems (mostly diarrhea, vomiting and loss of appetite), in 7.9% of cases antibiotic therapy was administered to treat and suppress non-specific symptoms (apathy, fever, pain). 11.6% of antibiotics were administered for the prevention and treatment of secondary bacterial infections in postoperative care, treatment of wounds and inflammation, as well as for associated bacterial infections in multisystemic viral diseases (typically panleukopenia).

**Figure 1:**
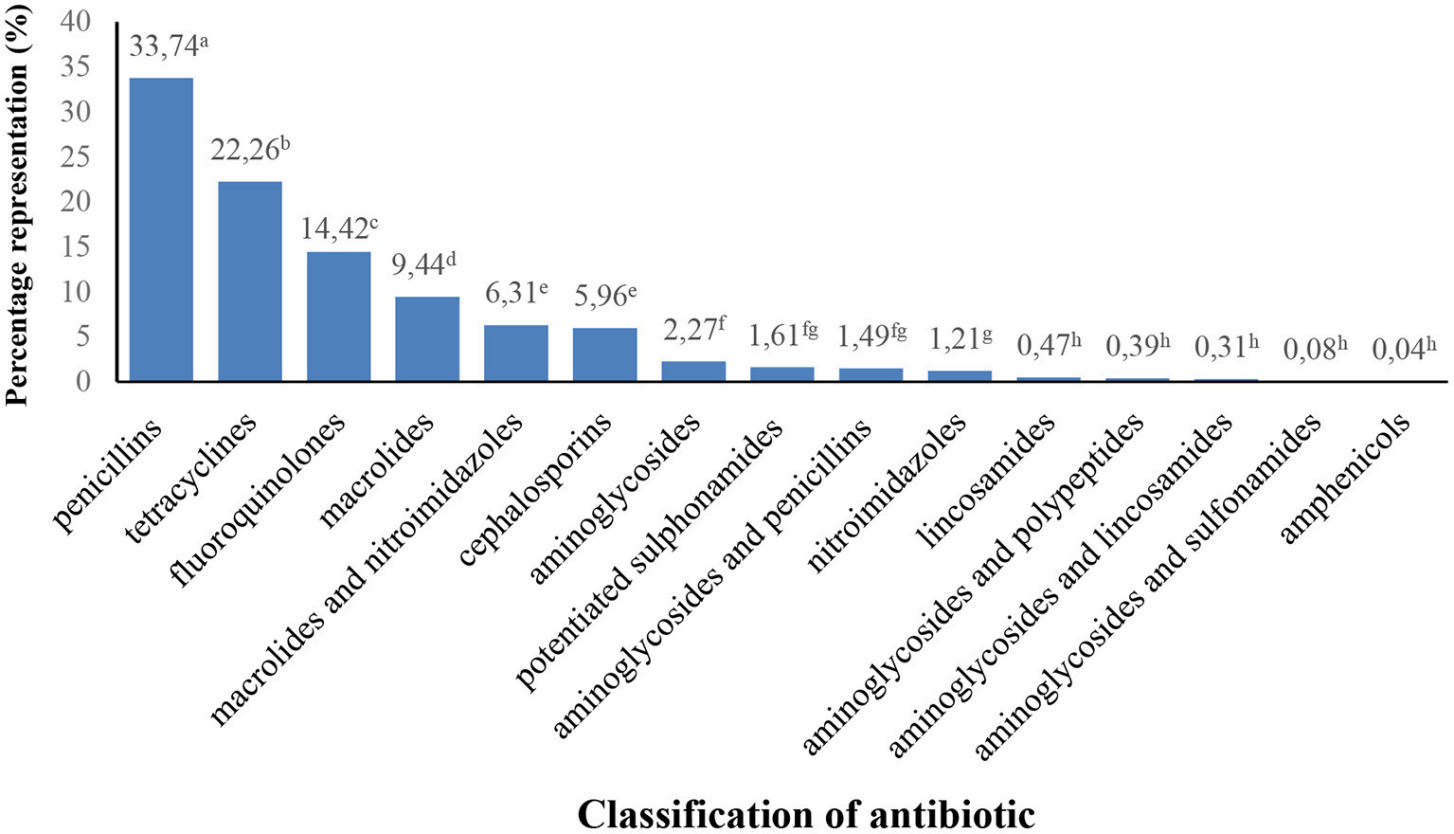
Number (%) of administered antibiotics according to the classification of the active substance. ^a−h^Chi-square test - percentages with different superscript letters differ (*p* < 0.05).

In terms of the spectrum of activity, antibiotics were divided into three categories (Table 8). Broad-spectrum antibiotics have been administered to cats most often. In terms of classification of antibiotics according to the active substance (Figure 1), the most used antibiotics were penicillins (amoxicillin in combination with clavulanic acid [838 administrations], benzylpenicillin [17 administrations] and ampicillin [6 administrations]), tetracyclines (doxycycline [568 administrations]) and fluoroquinolones (enrofloxacin [182 administrations], pradofloxacin [95 administrations], ofloxacin [50 administrations], marbofloxacin [38 administrations], moxifloxacin [2 administrations] and ciprofloxacin [1 administration]).

**Table 8:** Distribution of antibiotics used according to the spectrum of activity.

**The spectrum of activity of antibiotics**	**Number of administrations**	
	***n***	**%**
Broad-spectrum	2,084	81.66^a^
Medium to broad-spectrum	401	15.71^b^
Narrow-spectrum	67	2.63^c^
Total	2,552	100.0

a,b,c Chi-square test - percentages in the same column with different superscript letters differ (*p* < 0.05).

Following antibiotics, the second most often administered medicaments were those with antiparasitic effect (284 administrations, 7.63%). This number represents the number of antiparasitic products administered to cats in addition to the preventative treatment against internal parasites upon arrival at the shelter. Products to treat roundworms, flatworms and coccidia were administered to the cats 122 times (the active substances of these products were fenbendazole [87 administrations], toltrazuril [32 administrations] and emodepside in combination with toltrazuril [3 administrations]), ivermectin-based products to treat ear mites were administered 114 times. Therapeutic products containing fipronil for flea and lice elimination were administered 47 times. A medicament affecting both internal and external parasites (combination of fipronil, metoprene, eprinomectin and prazinquantel) was administered only once. In terms of statistical significance, the number of administrations of products for endoparasite elimination (flatworms/roundworms and coccidia) was significantly lower (*p* < 0.05) than the number of administrations of products used for ectoparasite elimination (ear mites, fleas and lice) (122 and 161, respectively).

Complementary and protective substances were administered 194 times (5.21%) and comprised of probiotics (administered four times) and products based on vitamins and minerals (administered 190 times). In all cases, these products were administered to compensate for malnutrition, gastrointestinal problems or general weakness. Choleretics (administered 169 times, 4.54%, active substance methylphenoxypropionic acid) were administered to animals for the treatment of liver failure, loss of appetite, diarrhea, vomiting and as an adjuvant in the treatment of panleukopenia. For the treatment and prevention of vomiting, as part of antiemetic agents (administered 102 times, 2.74%), active substances maropitant (90 administrations) and metoclopramide hydrochloride monohydrate (12 administrations) were used. Non-steroidal anti-inflammatory drugs (administered 101 times, 2.71%, active substances: tolfenamic acid [53 administrations], meloxicam [37 administrations], carprofen [5 administrations] diclofenac [5 administrations]) were most commonly used for the treatment of fever, postoperative pain and inflammation of the musculoskeletal system.

## Discussion

### Assessment of the health records of cats in shelters

The lack of data on morbidity of shelter cats in scientific literature is surprising, as such information is crucial for understanding the health issues and principles of animal treatment in shelters. Previously published studies have focused mainly on the mortality of cats in shelters and the risk factors associated with it (20–23).

The results of our study show that more than half of all cats whose records were analyzed had at least one health record made during their stay in the shelter. More than half of the cats, therefore, suffered from deteriorated health during their stay in the shelter that had to be treated by administering a medicament with a therapeutic or supportive effect.

The analysis of the impact of age on the number of cats' health records showed that within the age categories, the number of cats without health records and with health records differed significantly only in kittens younger than 6 months and cats older than 8 years. In both of these age categories, the number of cats with records was statistically significantly higher than the number of cats that were considered healthy (i.e., no health record was found). The highest number of health records was found in the youngest age category of cats, the same result was found in the number of health records related to the administration of medicaments and other supportive products. Age affects the risk of developing health issues, older cats and kittens are the most vulnerable category in terms of frequency and severity of diseases (24). The high morbidity of kittens is related to their immune system not being fully developed (25) and the overall poor health upon admission to the shelter, which according to Levy et al. (26) concerns feral cats to a greater extent than those who had received human care. In kittens, the highest mortality rate was also found in a study that monitored factors increasing the risk of mortality in shelters in the Czech Republic (22).

In 70.8% of cats, the medicaments and other supportive products were administered repeatedly. It is likely that the percentage of cats receiving treatment depends to a large extent on the approach of the particular facility. Some shelters choose the option of euthanasia in cases where another facility would apply treatment, even in spite of uncertainty of success (14). The treatment options are limited by available funds and staff, they also depend on the time, experience and knowledge of the shelter caregivers and veterinarians. The availability of particular drugs, equipment as well as the tradition of providing veterinary care at the level of the facility, region, or country complement the list of factors that affect the approach to treatment.

The number and type of medicinal products administered depends to a large extent on the condition of the individuals admitted to the shelter - e.g., severely injured animals will need a higher level of care combined with higher consumption of analgesics. The shelters that provided the cats' health records for the purpose of this study had not limited admissions only to a specific group of animals; animals from varying sources with different health histories were admitted to these facilities. Both facilities employed a no-kill policy. Neither shelter, however, used tools (evaluation scales, protocols) to evaluate the progress or regression of the applied treatment or any welfare protocol. The use of assessment tools may facilitate decisions concerning the continuation of treatment or euthanasia of animals.

In addition to the treatment of newly admitted animals, the number of therapeutic interventions also depends on the level of health of animals already housed in the shelter. The cat population in the shelter changes dynamically, admitted individuals differ in terms of their immunity and disease status, and the shelter environment creates pressure on animals and is stressful for them. One of the consequences of stress is suppression of the immune system function and the development of infection or its reactivation (27). Stress increases the risk of reactivation of the feline herpesvirus (28, 29), the risk of feline coronavirus (FCoV) shedding (30) and the development of infectious peritonitis (31), which is characterized by high animal mortality. Stress-induced increase in glucocorticoid levels is thought to be responsible for suppressing cell-mediated immunity, leading to increased FCoV replication (31). The claim that the shelter environment may have a negative effect on animals in the form of deterioration of health was also supported by our earlier study, in which we focused on the monitoring of health indicators of shelter cats' welfare over time. During the stay in the shelter, 41.6% of cats deteriorated in health (10).

In our study, the LOS of cats in the shelter correlated with the number of health records found. Cats for which no record was found stayed in the shelter until adoption for a significantly shorter time compared to animals for which health records were found. This finding is not surprising, the health condition is a factor influencing the adoption potential of animals. The phenomenon of preference of animals without health problems was also reported in studies aimed at the analysis of factors increasing the probability of adoption in dogs (13, 32). When comparing the LOS between adopted cats with health records and adopted healthy animals, age did not play a role only in cats over 8 years of age. It can, therefore, be concluded that the LOS until adoption of old cats was not affected by their health condition. Those willing to adopt an older animal probably expect the possible occurrence of health problems or accept these problems and decide to provide the animals with a home despite them. In our study, cats over 8 years of age stayed in the shelter until adoption for the longest time among all age categories. The fact that older cats are not as attractive from the point of view of adoption as younger individuals is also corroborated by the results of other studies (33, 34). Many potential adopters are likely aware of the risk of deterioration of health and the associated increase in financial costs for its treatment, but the advantage of adoption of young animals also lies in other aspects, e.g., young animals look more attractive and possess a high degree of adaptability and playful behavior. These factors have been described as important for potential new owners in the study by Sinn (35).

### Spectrum of administered medicaments and other supportive products

The results of this study showed that among drugs administered to shelter cats, antibiotics were used most often (68.57%). Similarly, Buckland et al. (36), who monitored the use of antibiotics in veterinary practice in the United Kingdom, reported that 21% of the total number of treated animals (594 812 cats) were administered antibiotics. Buckland et al. (36) found that 42% of dogs and cats were administered antibiotics repeatedly, which is also consistent with our findings (repeated administration was recorded in 62.07% of animals).

The basis for the success of antimicrobial therapy is the appropriate selection of antimicrobials with the desired spectrum of activity. A precise diagnosis is a necessary prerequisite (37). However, in a shelter environment it is often not possible to take samples for cultivation and sensitivity testing for each case. Collection of a sample, sending it to a laboratory for diagnostic purposes and obtaining the required information take at least 2 to 3 days. Laboratory testing can increase the cost for the entire treatment and shelters may not be willing to bear these additional costs (38). Shelter veterinarians, therefore, often use antibiotics empirically (1). Excessive use of antibiotics is a problem not only in human but also in veterinary medicine (39). Intensive medical care associated with the application of drugs increases the risk of nosocomial infections. In the animal population, there is an increase in the number of geriatric and immunosuppressed individuals which are susceptible to infections with multidrug-resistant pathogens, which may also pose a risk to the caregivers of such animals (40). The transfer of antimicrobial-resistant isolates has been already confirmed (41, 42). A higher risk of propagation can be expected in facilities with a higher concentration of cats, whether in households or in cat shelters. The use of antibiotics should be limited to situations where their administration is actually needed (17). A number of factors should be taken into account when selecting the appropriate antibiotic. If there is any doubt about the identification of the bacteria or if it is necessary to start treatment immediately, a broad-spectrum antibiotic should be used. However, once the information on the antimicrobial susceptibility is available, antimicrobial therapy should be changed to the use of a narrow-spectrum substance which specifically targets the bacteria (43). The selection should be based on knowledge of bacterial structures, pharmacodynamics and pharmacokinetics (38). Antibiotic therapy may potentially alter the intestinal, skin or respiratory microflora of treated animals. Documented consequences of antibiotic treatment include excessive proliferation of intestinal bacteria, e.g., *Clostridium difficile* (44).

In our study, penicillins and tetracyclines were among the most commonly used antibiotics (penicillins constituted 33.74% of all antibiotics administered, teracyclines 22.26%). The third largest group of antibiotics administered were fluoroquinolones (14.42%). De Briyne et al. (45), who monitored the use of antibiotics in veterinary practices in European countries (Belgium, France, Germany, Spain, the United Kingdom and Sweden) report that penicillins (37%), tetracyclines (14%), third- and fourth-generation cephalosporins (14%) as well as fluoroquinolones (13%) were the most frequently prescribed in cats. Among the listed groups of antibiotics, the frequent use of third-generation cephalosporins is mentioned in several studies (36, 46, 47). Hardefeldt et al. (48) reported that 25% of all antimicrobials applied in cats consisted of third-generation cephalosporins - cefovecin. The high rate of use of third-generation cephalosporins may be due to its simple subcutaneous administration and a long dosing interval in particular products (e.g., 14 days for Convenia with the active substance cefovecin). In addition, the preference of this product may be related to the fact that oral application is not successful in cats, thus administration by injection is preferred (49). Cephalosporins, however, comprised only a minority (5.96%) of antibiotics administered to cats in the shelters we monitored.

The analysis of the cats' health records showed that with the intent of high effectiveness of treatment, antibiotics were used alone or in combination with corticosteroids or other active substances, according to the diagnosis. In order to increase the possible effectiveness, a combination of antibiotics was also utilized. Schmitt et al. (39) who monitored the use of antibiotics in veterinary practices in Switzerland, reported that aminopenicillins with other antibiotics (fluoroquinolones, first generation cephalosporins, tetracyclines, amphenicols, or third generation cephalosporins) were generally administered mostly for treatment of acute upper respiratory tract disease, lower urinary tract disease, and abscesses. However, in shelters we monitored, penicillins were mainly applied individually (33.74%), i.e., without other supporting antibiotics. The exception was the combination of penicillins with aminoglycosides (1.49%).

From the point of view of the spectrum of action of the antibiotics used in cats, the use of antibiotics with a broad spectrum of action is prevalent (81.66%) in our study. The preference in use of broad-spectrum antibiotics in cats was also reported by Mateus et al. (50). However, they draw attention to the fact outlined above - the preference of broad-spectrum antibiotics is risky from the point of view of the development of antimicrobial resistance. In our study, narrow-spectrum antibiotics were used only in 2.63% of cases. A higher frequency of use of narrow-spectrum antibiotics (18%) was reported by Hardefeldt et al. (48). These antibiotics have an effect on a limited spectrum of bacterial flora, and only on gram-positive, gram-negative or otherwise specifically selected genera of bacteria. Such targeted treatment has its advantages only in the case when the disease agent has been clearly identified.

Amoxicillin in combination with clavulonic acid was the most frequently applied broad-spectrum antibiotic. This substance is a first choice for empirical therapy in a number of indications. Empirical therapy is necessary when the patient's clinical condition calls for immediate intervention. Absence of a microbiological examination to identify the causative agent and the establishment of its sensitivity may lead to the ineffectiveness of the prescribed antibiotic treatment. Thus, broad-spectrum antibiotics should not be prescribed unless the veterinarian is certain of the risk or presence of a bacterial infection. Preventive administration of antibiotics can lead to the presence or worsening of non-specific clinical symptoms such as loss of appetite, diarrhea and vomiting. As a result, the primary cause of the disease can be covered up. Improperly administered antibiotics can also cause unwanted drug interactions and, in addition, promote the selection of resistant bacteria (51).

Apart from penicillins and tetracyclines, fluoroquinolones were also commonly used antibiotics in the shelters we monitored. Urinary tract infections and soft tissue infections are main indications for their use. However, the application of enrofloxacin in cats poses a risk for the development of retinal degeneration. A smaller risk is presented by pradofloxacin, followed by marbofloxacin and orbifloxacin. The presence of renal disease may increase retinal degeneration. For this reason, fluoroquinolones should be only used in treatment of severe or recurrent infection diseases. Indications should be supported by previous results of culture and susceptibility tests (52).

In our study, more than half of all antibiotic administrations were recorded due to respiratory tract infection treatment, which is one of the most common problems in shelter cats (53). Respiratory diseases are a common cause of morbidity in cats as their management is complicated by a high concentration of animals and immunosuppression due to stress (54). However, in addition to the bacterial agents (*Chlamydophila felis, Bordetella bronchiseptica*, and *Mycoplasma felis*), symptoms are also associated with the infection of feline herpesvirus (FHV) and feline calicivirus (FCV) [e.g., (55)]. According to Pedersen et al. (56), about 4% of cats excrete FHV when admitted to the shelter, in the case of FCV it is about 11% of cats. After the 1st week spent in the shelter environment, the infections are reactivated; the excretion of FHV increased to 52%, FCV was excreted by 15% of cats (56). In the case of FCV, vaccination provides a good protection against its acute oral and respiratory form, but in most cases does not prevent the infection and excretion of the virus. Moreover, none of the vaccines protects against all field strains of FCV (57). Similar to FCV, vaccination against FHV provides protection against the development of clinical symptoms and reduces virus excretion within 1 week after administration (58) but does not provide animals with complete protection. Nevertheless, vaccination of shelter cats against both FHV and FCV is recommended (59). Both shelters monitored in our study routinely vaccinated animals against calicivirus, herpesvirus, and panleukopenia upon admission to the shelter if they had reached the required age.

For the treatment of upper respiratory tract infections, antibiotics should be used primarily for the treatment of infections caused by *Mycoplasma Felis, Chlamydophila felis* and *Bordetella bronchiseptica*, where the first choice of antibiotic is doxycycline. Antibiotic therapy can also be used wherever bacterial pathogens are evidently present, e.g., in cases of chronic rhinitis and sinusitis. In these cases, amoxicillin in combination with clavulanic acid is a suitable choice (1). However, fluoroquinolones (enrofloxacin) (46) or ampicillin (60) can also be used to treat respiratory diseases.

Another reason for the application of antibiotics was the prevention and treatment of secondary bacterial infections as part of postoperative care, wound care and inflammation. Antibiotic therapy has also been introduced in the case of development of bacterial infection associated with multisystemic diseases of viral nature and in the case of non-specific symptoms. According to De Briyne et al. (45), the most common indications for the use of antibiotics in cats are skin diseases (42%), respiratory diseases (24%), urinary tract infections (16%) and periodontal diseases (14%). The use of antibiotics for skin disease treatment is generally high and has been reported by various authors (39, 61, 62). Cefovecin belonging to third generation cephalosporins is often used to treat skin trauma and abscesses (47). However, the results of our study did not show the high level of administration of antibiotics for skin diseases in the monitored shelters.

Gastrointestinal problems were a significant group of health problems for which antibiotic therapy was chosen in the monitored shelters (28.4%). These may indicate a problem localized directly in the digestive tract, but gastrointestinal symptoms may accompany a multitude of other pathological changes that are manifested by many symptoms. In our study, diarrhea, vomiting and loss of appetite were among the most commonly occurring. It is important to mention that bacteria are rarely the cause of gastroenteritis in cats. For that reason, antibiotic treatment is undesirable in most cases. The occurrence of acute diarrhea is often self-limiting and in case of absence of serious symptoms, it usually resolves without veterinary intervention. However, antibiotics are often prescribed to dogs or cats in the presence of acute diarrhea (63).

Diarrhea and vomiting are typical symptoms of, for example, Carnivore protoparvovirus 1 infection, which causes feline panleukopenia. Although the pathogen is of viral origin, in this case the administration of antibiotics is indispensable because the sepsis associated with the translocation of bacteria in the gastrointestinal tract and immunosuppression are the primary cause of death. A suitable choice of therapy is amoxicillin with clavulanic acid in combination with marbofloxacin (64). According to the available literature on the treatment of gastrointestinal diseases, it is generally appropriate to use, among others, amoxicillin with clavulanic acid, furthermore also doxycycline (65). The use of amoxicillin with clavulonic acid in the treatment of acute diarrhea was discussed by Werner et al. (66) in dogs. The results of their study showed that the effect of these antibiotics on the treatment of acute diarrhea has no benefit and increases the risk of *Escherichia coli* resistance. Stavroulaki et al. (65) reported negative effect on the microbiome of cats when using amoxicillin/clavulanic acid or doxycycline. These antibiotics also slowed the development of the microbiome in growing cats. Werner et al. (66) confirmed that the only reason to use antibiotics in cases of acute diarrhea is the risk of sepsis, which is in accordance with international recommendations (67). Veterinarians should be aware of the risks that antibiotic treatment can bring in cases of gastroenteritis and should apply them only according to the conclusions of the clinical and laboratory examination itself (the signs of sepsis: left shift, toxic changes in neutrophils) (68, 69).

Aside from pathogens of viral, bacterial and parasitic origin, stress is a significant contributor to the development of gastrointestinal problems. One of the consequences of stress is the disruption of the intestinal barrier, which causes an increase in its permeability and a local inflammatory response (70). However, diarrhea resulting from inflammatory processes may also occur due to a change in diet after admission to the shelter. German et al. (71) reported diarrhea in 11.9% of shelter cats, Andersen et al. (72) found the occurrence of diarrhea in up to half of all cats in the shelter. In our study, gastrointestinal problems were addressed by the administration of other types of products (mainly choleretics, antiemetics, antidiarrheals and additives and protective agents) in addition to antibiotics. Complementary and protective substances in the form of probiotics and products based on vitamins and minerals were administered to the animals in order to improve immunity and alleviate gastrointestinal symptoms. The use of probiotics may reduce the occurrence of diarrhea in shelter cats (73).

After antibiotics, the second most numerous group (7.63%) of administered medicinal products were antiparasitics. Products for the elimination of endoparasites were administered routinely to animals upon admission to the shelter, but in some cases repeated administration was necessary during the stay. Preventive administration of anti-endoparasites is likely to be related to the finding that anti-ectoparasites were administered in more cases compared to anti-endoparasites (4.33 and 3.28%, respectively). Among endoparasites, the occurrence of helminths and coccidia was addressed using products containing fenbendazole, toltrazuril and emodepside in combination with toltrazuril. Tull et al. (74) monitored the occurrence of endoparasites in a cat shelter in Estonia and found that 47.6% of cats were infected. In their study, cats were mostly infected by *Toxocara cati* (36.6%), one of the most common zoonotic intestinal parasites. Other parasites found in cats included coccidia (*Cystoisospora spp*.; 12.4%) and tapeworms (*Taeniidae gen. sp*.; 4.1%). An Italian study by Sauda et al. (75) found a parasite prevalence of 22%; up to 19.7% of shelter cats were infected with parasites with zoonotic potential. Sauda et al. (75) found a higher prevalence of protozoal infections compared to helminth infections. The parasites found were *Giardia duodenalis* (10.6%), *Toxocara cati* (9%), *Cystoisospora felis* (3%), *Cystoisospora revolta* (2.3%), *Cryptosporidium spp*. (1.6%), *Aonchotheca putorii* (0.75%), *Tritrichomonas fetus* (0.75%) and *Strongyloides sp*. (0.75%). The data on occurrence of individual species of parasites were not available in the health records monitored in our study.

In the monitored shelters, ectoparasite as *Otodectes cynotis* was treated using a product with ivermectin (3.06%), and fleas and lice using fipronil (1.26%). *Otodectes cynotis* is highly contagious and poses a risk in an environment with a high concentration of cats. The parasite may also occur in animals with unchanged appearance of the ear canal (76), therefore it is desirable to perform a swab from the ear canal with subsequent examination under a microscope. Genchi et al. (77) reported a prevalence of *Otodectes cynotis* of 9.8% in cats originating from feral cat colonies, shelters and private households. A higher incidence rate (19.3%) was found in free-roaming cats in Oklahoma, USA (78). The cats' age of under 12 months and access to an outside environment were factors increasing the risk of parasite infestation. Young cats are thought to exhibit playful behavior and are likely to come into contact with other cats, which predisposes them to infection (77). In the absence of antiparasitic treatment, in addition to the local effect on the ear canal, there is a risk of parasite spreading to another body parts (79). Fleas are also prominent among cat ectoparasites (80). The parasite induces allergic reactions and may transmit bartonellosis or *Dipylidium caninum*. In the free-roaming cat population, the incidence of *Ctenocephalides felis* can go as high as 71.6% (78).

Quarantine of newly admitted individuals is important not only in an effort to minimize the transmission of bacterial and viral agents in the population, but also to rule out or eventually eliminate internal and external parasites. This is consistent with the findings of Tull et al. (74), who reported a lower intensity of infection in quarantined cats (held 1–14 days in the shelter) in comparison to cats after quarantine that were kept individually or in group housing.

Due to the need to protect animals against the introduction of parasites into the population, shelters usually proceed to administer antiparasitics without examination, which was also the case of the shelters monitored in our study. At this time, resistance to feline endoparasites is not a problem, but there have been reports of insufficient effectiveness of anti-ectoparasitic products. Such cases are primarily characterized by a shorter period of effectiveness (81).

## Conclusion

Although a relatively high number of animals was found with morbidity-related records (61.4%), mortality in the monitored shelters was relatively low (14.1%). The low number of euthanized animals as well as unassisted deaths may be partly hinting at the effectiveness of therapeutic procedures and to the low admission rates of animals in life-threatening conditions or at the capacity to manage them. Although reducing mortality is desirable to the greatest possible extent and is a common goal of shelters, efforts should also be made to prevent, cure or at least mitigate the negative effects of deteriorated animal health. Good health of animals is one of the key aspects of welfare. Particular attention should be paid to the youngest age category of animals for which the number of health records found was the highest.

Furthermore, the health of cats is a factor that affects the LOS in the shelter until adoption. However, the presence or absence of health problems does not seem to play a role in the case of old cats (in our study, cats over 8 years of age) and the reasons for this finding should be further investigated. Regardless of this result, shelters should generally strive to reduce the LOS by maintaining animals in the best possible condition.

Antibiotics were found to be the most commonly used drugs in the treatment of shelter cats in our study. This finding reflects the nature of the most common problems occurring in the shelter cat population (respiratory tract infections and gastrointestinal problems). Antibiotics should be used in a targeted manner and only when necessary, otherwise there is an increased risk of resistance. Measures to limit the use of antibiotics should be set up in shelters. A possible approach is the proper training of veterinary staff and the supervision of the provided veterinary care. Raising overall awareness of antibiotic overuse could contribute to a change in thinking about these substances.

Antiparasitic agents constituted another significant category of products probably due to the fact that the shelters admitted animals of various origin, including stray cats. According to the previous studies, the rate of infestation with parasites is elevated in stray cats.

This study has two main limitations. The first limitation concerns the number of shelters from which data were obtained for analysis. Based on the national legislation of the Czech Republic, shelters are not obliged to keep the health records on shelter animals, therefore many facilities do not have a system for archiving such data. This fact contributed to the selection of those facilities that systematically store this type of data. The second limitation is the fact that analyzed records did not include information on surgical procedures and treatment given to cats at veterinary clinics in case of their hospitalization. None of selected shelters has equipment that would allow animals to be hospitalized (in general, it is not common in Czech shelters to have such equipment within the shelter), thus if needed, the animals were transported to a private veterinary clinic for treatment. Data regarding products with a therapeutic or supportive effect that were administered to the animals during hospitalization were not recorded in the shelter's data storage system, thus they could not be obtained. We may also assume that the results of the study might be influenced by the specific conditions in the selected shelters (management, environment, staff, funding etc.).

Preventing the introduction and spread of diseases is extremely important in order to maintain animal health and welfare and setting up appropriate procedures is one of the most challenging tasks that shelters have to face. From the point of view of the material equipment constituting the shelter environment, the facilities should find a compromise between the need for frequent sanitation and the behavioral needs of animals, which include opportunities for comfortable rest, hiding, observation from elevated places, etc. From the point of view of the effectiveness of sanitation, it is necessary to take into account the adequacy of the chosen disinfectant and its mechanism of action. Shelters should also aim to eliminate stress in animals as it is related to health disruptions. Since respiratory diseases and gastrointestinal problems are a frequent consequence of overcrowding, shelters should reevaluate the possibilities of making changes regarding management and the number of admitted individuals.

## Data availability statement

The original contributions presented in the study are included in the article/supplementary material, further inquiries can be directed to the corresponding author/s.

## Ethics statement

Ethical review and approval was not required for the animal study because no experimental procedures were performed. The cats were housed and provided with care by the shelter personnel in accordance with the current animal welfare and veterinary legislation.

## Author contributions

Conceptualization and methodology: VVo, EV, and MK. Data curation: VVo and EV. Supervision: EV and VVe. Writing—original draft: VVo, MK, and LT. Writing—review and editing: EV, VVe, MK, and LT. All authors contributed to the article and approved the submitted version.

## Funding

This study was supported by ITA VETUNI (Project No. 2022ITA21).

## Conflict of interest

The authors declare that the research was conducted in the absence of any commercial or financial relationships that could be construed as a potential conflict of interest.

## Publisher's note

All claims expressed in this article are solely those of the authors and do not necessarily represent those of their affiliated organizations, or those of the publisher, the editors and the reviewers. Any product that may be evaluated in this article, or claim that may be made by its manufacturer, is not guaranteed or endorsed by the publisher.
